# Knowledge in Transition in Healthcare

**DOI:** 10.3390/ejihpe10030054

**Published:** 2020-07-17

**Authors:** Maria José Sousa, Francesca Dal Mas, Alexeis Garcia-Perez, Lorenzo Cobianchi

**Affiliations:** 1 Instituto Universitário de Lisboa, Business Research Unit, 1649-026 Lisbon, Portugal; 2 Lincoln International Business School, University of Lincoln, Lincoln LN5 7AT, UK; fdalmas@lincoln.ac.uk; 3 Faculty Research Centre for Business in Society, Coventry University, Coventry CV1 5DL, UK; ab1258@coventry.ac.uk; 4 Dipartimento di Scienze Clinico-Chirurgiche, Diagnostiche e Pediatriche, Università degli Studi di Pavia, 27100 Pavia, Italy; lorenzo.cobianchi@unipv.it; 5 Dipartimento di Scienze Chirurgiche, Fondazione IRCCS Policlinico San Matteo, 27100 Pavia, Italy

**Keywords:** healthcare organization, knowledge in transition, static knowledge, dynamic knowledge

## Abstract

Organizations are challenged by the need to transform Dynamic Knowledge, embedded in each worker, into Static Knowledge, rooted in factual documental information. However, innovation and knowledge creation seem to be facilitated by the personal knowledge and life experiences of people, which appear to be dynamic. The tensions between Dynamic and Static Knowledge in facilitating the transfer and sharing of knowledge arise as compelling research as well as practical topic for organizations. Our paper aims to investigate such tensions by employing a case study. We decided to deepen such dynamics in the healthcare field, given its importance for business and society. In more detail, we analyzed one Emergency Room (ER) department through a series of interviews. Our findings highlight the importance of the right balance between Static and Dynamic Knowledge. On the one hand, the healthcare organization recognized the need to incorporate knowledge into practical and tangible instruments. On the other hand, the flows of Dynamic Knowledge must be fostered through a culture of knowledge translation and sharing, and the development of soft skills.

## 1. Introduction

This article aims to understand the tensions between two opposite forces: Static Knowledge and Dynamic Knowledge, and the mechanisms of knowledge transition in healthcare organizations. It will present a literature review about knowledge and its nature, and it will analyze the role of the health professionals in knowledge transition processes within organizations.

A fundamental part of organizations’ knowledge is dynamic [1], rooted in each worker—the so-called individual knowledge [2]—based on their work and life experiences [3]. Another essential part of organizations’ knowledge is static, embedded in documental information [4].

Dynamic Knowledge can be expressed in opinions, behaviors, ideas, and informal conversation, through workshops, communities of practice [5], and meetings of various kinds [6]. Static Knowledge is usually stored in reports, memos, document procedures, databases, wikis, and other types of organizational documentation [7].

Dynamic Knowledge should be stored in repositories so that it can become a substantial source of relevant information and expertise. However, knowledge flows much better under informal networks, assuming a dynamic nature, than through the hierarchical structure, where Static Knowledge has a more significant importance in the form of reports, memos, and other organizational documents.

Starting from this premise, our work wants to examine in more depth how these dynamics work in one sensitive field, that of healthcare. Healthcare is essential due to its contribution to the wellbeing of society [8,9]. At the same time, the healthcare sector is undergoing a relevant change [10], due to the introduction of always-new technologies [11], and protocols in surgery and care [12,13,14], the requests for more inpatient and outpatient services by an ageing population [15], and the need for accountability and transparency with a reduced budget [16]. In particular, we decided to examine how knowledge flows in the Emergency Room (ER), probably the most hectic department in hospitals [17]. At the ER, healthcare professionals need to cope with patients with a variety of different conditions, deciding whom they should assist first, which other departments to involve, which people to hospitalize or send home, and what to do in case of a global emergency (like the case of Covid-19) [18]. Using a case study approach, we carried on some interviews to map and deepen the knowledge dynamics, following the framework of Lopes at al. [19].

The article is the output of larger research being developed in one healthcare institution, as part of a project on competencies development and knowledge management processes. The whole research has the goal to identify the skills and knowledge forms of transition and to infer about future capabilities and new ways of knowledge transfer among health professionals. This first phase of the study’s purpose is to identify the current processes and competencies, to define practical implications on the definition of new strategies for health professional management in terms of their future development, aiming at more efficient and agile management practices in health institutions. The article will also contribute to the knowledge management theory in terms of addressing more robust concepts of Dynamic and Static Knowledge, emerging from the field. 

In this context, two research questions (RQ) were defined: 

RQ1: ‘What is the individual knowledge translated in the main competencies used by the health professionals of the ER to perform their jobs?’; 

and 

RQ2: ‘What are the main knowledge transition mechanisms used in the ER?’

The paper is structured as follows. First, it presents a literature review on knowledge transition, Dynamic, and Static Knowledge. Then, it highlights the methodology and the main findings and discussion. A conclusion paragraph ends the paper.

## 2. Literature Review

### 2.1. Knowledge in Transition Conceptualization

Knowledge in transition is a process of organizational innovation and needs to be modelled, structured, and partially formalized by the knowledge sharing process [20,21,22]. This idea is expressed in the research of Argote and Ingram [23], who conclude that knowledge sharing among workers represents a competitive advantage for organizations. They highlight that knowledge sharing is “the process through which the experience of one unit affects another” and argue that interactions involving workers allow more excellent knowledge sharing within organizations. They conclude that knowledge embedded in the interactions of workers and tasks potentiates the organization’s capability to innovate and be more competitive. Hansen et al. [24], Massaro et al. [25], and Jacquinet et al. [26] also emphasize the importance of the worker’s role in knowledge sharing activities. They consider the balance between the uses of technologies for knowledge sharing and transition activities versus relying on people to share knowledge through more traditional means. The transition process means codification through, i.e., information systems, opening up the possibility of large-scale reuse for organizations.

In contrast, a personalization approach invests more in facilitating conversations and the exchange of individual knowledge [27]. However, a primary aspect of knowledge sharing among individuals in organizations is trust, and Levin and Cross [28] pointed out its importance when they referred to the competence and trust among individuals in an organization that influences the link between them and the effective use of knowledge. Lee and Choi [29] also note that the lack of trust among employees is another critical barrier to knowledge sharing activities and posterior transition into organizational knowledge. To overcome such obstacles, organizational studies point out the importance of democratic and participative leadership as the main factor to enable a culture of knowledge sharing [30,31,32]. In a culture of knowledge sharing, the transition process of individual knowledge into organizational knowledge may be facilitated with the use of a common and shared vocabulary. Cummings [33] reinforces that idea, considering the influence of structural diversity on work group performance, meaning that, when members of diverse workgroups are capable of sharing external knowledge to the group, their performance improves, and the organizations become more innovative.

### 2.2. Dynamic Knowledge versus Static Knowledge

Most knowledge in organizations is dynamic, rooted in each worker, and a small part is static, embedded in documental information [34]. Dynamic Knowledge should be stored in tangible repositories so that it can become a substantial source of relevant information and expertise [35], as reported in Table 1 [30,36]. 

A similar distinction, in the literature, refers to explicit and tacit knowledge [37]. While explicit knowledge is the most basic form of knowledge, when data is stored, processed, organized, and structured, allowing sharing in an easier way, tacit knowledge is possessed by people, and it is garnered from personal experience and contexts [38]. Converting tacit knowledge into an explicit one is one of the biggest challenges for organizations [39], as it contributes to the competitive advantage, fostering knowledge transfer and sharing [40]. 

When referring to the healthcare sector, communities of practice (CoPs) can result in effective knowledge sharing among CoP participants, creation of new knowledge, and improvement of practice [41]. Telemedicine and new technologies can help healthcare professionals in sharing knowledge effectively [42], also considering the amount of information to be managed and shared [43]. The use of new technologies also involves social media networks [44]. Knowledge sharing may also include behaviors like best practices, mistakes, and feedbacks [45]. 

Workers that participate in the resolution of specific problems [31] develop strategies that can be learned by other workers and be applied in different situations—capturing knowledge shared in real-time—this is a process of capturing and reusing Dynamic Knowledge. Effective capture and reuse of Dynamic Knowledge within the organization, such as the capture of personal knowledge, may be achieved using a common and shared vocabulary. This can be promoted by the creation of a culture of knowledge sharing [36].

## 3. Methodology

In the paper, we employ a qualitative case study approach [46]. Qualitative methodologies enable investigators to uncover and understand the relationships among different variables, even when situations are compared, and to justify the influence of the social context [47]. Moreover, case study methods seem to fit the context better when a how or why question is asked on contemporary events where the investigator has no control [46]. Last but not least, case studies grant a deepening of a real-world case [48]. To ensure transparency [49], in the following subparagraphs, we explain the research context and the data collection and analysis.

### 3.1. Research Context

We picked one public healthcare organization located in the center of Portugal, selecting the ER as one of the most critical departments in terms of knowledge needed, and the necessity of agile and quick decision-making [17]. The healthcare organization is public, and it is also a University institution, ranked as one of the biggest health infrastructures in the country. The hospital was chosen as it is part of a larger project about health innovation, showing thus excellent innovative capabilities.

### 3.2. Data Collection and Analysis

Data were collected through three group semi-structured interviews (Appendix A), with at least two professionals in each group, meaning a total of 7 interviews. The healthcare professionals that participated in the research had the role of Operation Assistants, Emergency Room Nurses, and Emergency Medical Technicians. The research approach is based on a case study. The ER was the context studied, as it was the first department under the analysis of the research, and it represents the heart of the health institution. The interviews were conducted in a meeting room, and after the end of the working shift of the health professionals. The information was collected with the help of the tables of competencies (Table A1, Table A2 and Table A3), and also notes regarding the other questions were taken. As a qualitative study, the number of interviews is made by convenience sample, according to the workload and the shifts of the health professionals. The duration of each interview was about 50 minutes. The Emergency Medical Technicians and the Operations Assistants were male, while the Emergency Room Nurses were female. The goal of the interviews was to understand how the knowledge was translated and transferred among the professionals. The interviews were coded and categorized, and then the information was analyzed according to those categories, with an excel sheet that was used to code and identify the categories. 

For collecting data, researchers have taken field notes to register not only the comments from the different health professionals, but also their perceptions. Field notes were taken during visits to the health institution and informal conversations with the health professionals, with their acknowledgement, as the research is authorized and was communicated to the organization by the board of the health institution. The main goal of the interviews was to collect individuals’ opinion about their competencies and the knowledge transition processes.

## 4. Findings 

### 4.1. Static and Dynamic Knowledge Transition Processes in the Filed—the Perceptions of the Health Professionals

Knowledge shared in everyday work in the healthcare organization is a process of transition from individual knowledge (Dynamic Knowledge) to organizational knowledge (Static Knowledge) [30].

Static and dynamic knowledge are, by nature, extremely difficult to translate, not only because dynamic knowledge is, to some extent, individual and tacit—acquired during life experiences and learning processes; but also, because static knowledge is explicit in research, books, and organizational routines, practices, and contexts. All of the knowledge needs to be learnt and is the base of competencies development, to perform a job position or even to grow from a personal perspective. 

According to their nature, Dynamic and Static Knowledge have completely different characteristics and are shared in completely different ways, needing different kinds of competencies for its effective translation. Static Knowledge is regarded as objective, free from individual subjectivity, while Dynamic Knowledge is highly subjective, being embedded within the cultural values and assumptions of those who possess and use it. 

Knowledge translation requires extensive and direct social interactions between professionals, as it is during such processes that the dynamic component of knowledge can be shared [50,51,52,53]. This is confirmed by Nonaka and Takeuchi [54] who have crystallized the idea that it is the interaction of people that leads to the creation of new knowledge, in their “knowledge spiral.”

Next, we will discuss forms of knowledge translation supported by the base idea of Nonaka and Takeuchi’s Knowledge Spiral. However, we will not use the categorization of the model because we think that the processes of creation and use/share of knowledge cannot be separated. It is a dynamic process that blends all forms of knowledge sharing.

Transferring dynamic knowledge requires specific competences of interaction because it represents knowledge that people possess, but which is inexpressible and incorporates both physical skills and cognitive frameworks. In the health institution (our case study), the transfer of knowledge between the health professionals is based on long years of experience, especially when new professionals arrive at the institution. This knowledge is shared through an extensive amount of social interaction and face-to-face communication. One ER nurse declared:

“There are some concerns about the information and about procedures and problem-solving processes, especially because of new workers. We have some routines for their integration, and one of these routines is based on a coaching process that occurs with a more experienced colleague that knows the work procedures, and helps the new workers to develop their knowledge and competencies in the first weeks, showing them what to do and how to do it. “

To make this process of knowledge translation effective, the focus was the creation of a trustworthy atmosphere, making all the professionals more participative and more involved. One Operations Assistant stated: 

“The health supervisor participates in the integration of new workers, helping with the coaching process. When some doubts arise, the new health professionals consult the colleagues and the supervisors.”

These interactions among workers and managers are also important when they share and translate explicit knowledge, because of the inherent ambiguity of language and because people have different cognitive frameworks, creating scope for differing interpretations. 

Tsoukas [55] gives validity to this idea when he suggests that tacit knowledge and explicit knowledge are inseparable and are mutually interconnected. Without a tacit understanding of the language in which explicit knowledge is written or the grammar and syntax used to structure it, any text will appear as a somewhat random series of letters, numbers, and images. Thus, there is no such thing as fully explicit knowledge, as all knowledge is ‘either tacit or rooted in tacit knowledge’ [56]. Alternatively, to state it succinctly, ‘all knowledge has tacit dimensions’ [57].

In this context, the coaching process assumes here a critical role because no matter how explicit and well defined the procedures and routines are, there will always be some element of ambiguity or uncertainty, creating a need for analysis and comprehension. After all, ‘knowing’ and ‘doing’ are two inseparable processes, and knowledge development occurs on an ongoing basis through the routine activities that workers undertake, based on the competencies they have.

In our case study, these ideas can be illustrated through the process of applying the Operations Assistants knowledge with the help of the Emergency Room Nurses and the Emergency Medical Technicians, together with experimentation, observation, and dialogue techniques, which allow the adaptation of existing knowledge to new and novel situations. This represents an important and undervalued source of learning, and the processes of learning by observing are crucial for the new workers. They learn through socialization, observation, and practice. One Emergency Medical Technician declared:

“The instruction sheets of procedures and the now existing competencies tables represents important information, which can be used by the new Operations Assistants, and the new Emergency Room Nurses. But first, they learn with the supervisors how to perform the work routines.”

Davenport and Prusak [22] confirmed through their studies that the translation of knowledge can be made by formalized transfer mechanisms and also informal exchanges. The formalized transfer methods include documents, databases, and Intranets. Informal exchanges refer to the more casual events that usually take place face to face, such as a conversation. Applying this to our study we can state that to translate knowledge as rules, procedures, and routines, several techniques can be applied, like procedures sheets; knowledge databases for emergency problems and solutions, and others repositories where information and documents are stored. Such documents can be reused and shared, for example, regarding operational specifications, manuals, and other information about medical procedures. In respect to Dynamic Knowledge, it flows during the operations in the ER, when the health professionals gather to save a patient’s life, most of the time a situation which requires a mix of static knowledge—correspondent also to organizational knowledge, and Dynamic Knowledge—correspondent also to individual knowledge. 

#### 4.1.1. Individual Knowledge 

Within the knowledge sharing processes, our interviews highlighted how workers continuously refine their organizational, technical, cognitive, and social competencies. To identify the skills related to individual knowledge use and sharing, we adapted Lopes et al. [19] typology of competencies based on the typology of Le Boterf [58] and Green [59].

The following Table 2 reports our results concerning the individual knowledge, following the above mentioned framework, and responding to RQ1: What is the individual knowledge translated in the main competencies used by the health professionals of the ER to perform their jobs?

#### 4.1.2. Organizational Knowledge 

**Figure 1:**
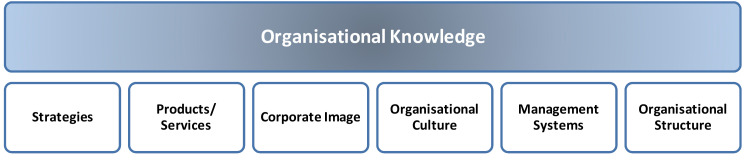
Organizational knowledge.

A large portion of organizational knowledge is connected to information repositories in the form of stored documents across the company (Static Knowledge). This knowledge was initially rooted in the workers’ heads (Dynamic Knowledge). Still, as a relevant organizational asset, the organization feels the need to create mechanisms to store such knowledge in different formats, such as text files, presentation slides, spreadsheets, email messages, and wikis, among others. These documents are a common source of information about the organization, and represent the organizational knowledge, embedding the strategies, products or services, corporate image, management systems, and the organizational structure, as reported in Figure 1.

This knowledge is most important for the organization and its continuous improvement, as it allows us to define the future direction of the healthcare organization.

### 4.2. Knowledge Transition Mechanisms 

The case study main findings highlight that all the professionals participated in the resolution of specific problems and developed strategies that could be learned by other healthcare professionals. Such procedures could be applied in other areas or departments of the health organization, capturing knowledge shared in real-time. In essence, this involves a process of knowledge in transition from Dynamic to Static knowledge.

**Figure 2:**
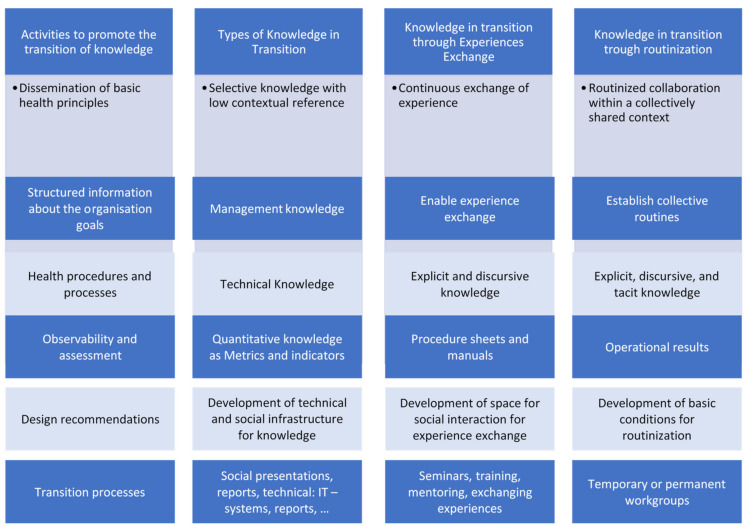
Knowledge transition mechanisms—case study.

The following Figure 2 presents the main findings of the research, structured by the dimensions: Activities to promote the transition of knowledge; types of knowledge in transition; knowledge in transition through experiences exchange; knowledge in transition trough routinization, responding to RQ2: What are the main knowledge transition mechanisms used in the ER?

The table above shows that the basic principles of the organization are generally transmitted with low contextual reference, but as a continuous exchange of experience and also with work routines.

The health organization’s goals are usually easily transmitted, enabling experience exchange, and establishing collective routines. Focal knowledge, such as knowledge about products or services, is transmitted explicitly, in particular through instruction manuals or other kinds of documentation.

Observability and assessment are transmitted as explicit knowledge involving the operational results of the organization’s activity. Design recommendation is transmitted through technical and social infrastructure. Finally, the transition process is made through presentations, reports, and IT systems. 

Effective transition of Dynamic Knowledge within the health organization, such as the capture of specific knowledge, may be achieved using a common and shared vocabulary, and this can be promoted by the creation of a culture of knowledge sharing.

## 5. Discussion

Individual knowledge or Dynamic Knowledge underlies many possibilities for organizations when it is deeply embedded in its practices and procedures. It includes relationships, norms, values, and standard operating procedures, and it is very hard to detail, copy, and translate, in opposition to Static Knowledge, which is found in the manuals and procedures sheets. 

This research analyses the forces between these two dimensions of knowledge and tries to capture the mechanisms of knowledge translation, and the individual knowledge translated into competencies needed to perform the job activities of the participants in the study: Operations Assistants, Emergency Room Nurses, and Emergency Medical Technicians.

To operationalize the study, we used Nonaka and Takeuchi’s model on knowledge [54] as a framework in the analysis of the interviews, as a cyclic process involving four related activities: (1) Socialization, which is an interaction moving from tacit to tacit knowledge; (2) externalization, an interaction moving from tacit to explicit knowledge; (3) combination, an interaction moving from explicit to explicit knowledge; and (4) internalization, an interaction from explicit to tacit knowledge. However, not considering all these phases independently, but as an integrated process, and in this research, the externalization is the crucial activity that transforms individual knowledge into organizational knowledge, allowing the transition of knowledge. 

**Figure 3:**
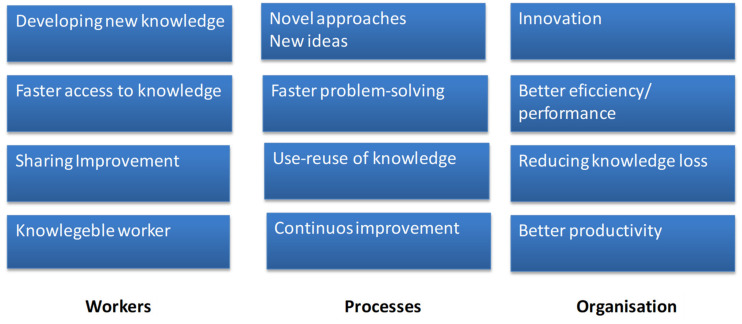
Knowledge transition benefits—case study.

In this context, health professionals may be involved in knowledge activities because of their intrinsic drive for learning, personal contentment, peer recognition, and self-actualization, in line with several studies in the subject that also confirm that these behavioral motives play a major role in the knowledge transition process, and in the individual knowledge and competencies development process, with major benefits for all the actors involved The following Figure 3 reports the main knowledge transition benefits. 

The benefits for health workers derived from more efficient processing of information and knowledge by, for example, eliminating the duplication of efforts or saving valuable time. The benefits for processes could be translated into benefits that can be expressed in terms of efficiency or effectiveness. Databases are a common example since they help to eliminate less efficient operations by reusing knowledge. The impacts on organizations affect some of the health institution key goals, such as productivity, performance, and innovation. Moreover, knowledge in transition can also be viewed as an innovation with the potential to generate new ideas, develop workers’ competencies, and create advantages for the health organizations.

## 6. Conclusions

Organizations are challenged to find ways to transform and translate Dynamic Knowledge into Static Knowledge, embedded in documental tools, to capture knowledge and allow the transfer and sharing of it among people and teams. However, Dynamic Knowledge, that flows and is shared in more informal ways, is relevant to enhance innovation and the creation of new knowledge. 

We decided to investigate the healthcare sector, employing a case study carried on in an Emergency Room department. In the ER, healthcare professionals face different situations and emergencies every day, and they must cope with the need to make decisions quickly. The need to rely on tangible tools and instruments is balanced by the necessity to use more intangible techniques and skills, including teamwork. Tensions emerge, as our investigation highlights the need for healthcare professionals to work employing both Static as well as Dynamic Knowledge at the same time. 

The call for a clear and formalized table of competencies to identify the crucial skills and tasks for each job position is balanced by the need to enhance a general culture of knowledge sharing and teamwork. Training may include not only the development of technical and hard skills, but also the enhancement of soft skills such as problem-solving. 

Our study stresses how the situation can be complex and subtle. Static and Dynamic Knowledge must coexist. Only their balance can help the successful development of an organization. We investigated one healthcare organization and, in particular, one particular department (the ER).

The need to translate the Dynamic Knowledge into formal and tangible tools, and at the same, the call for creative ways to enhance informal engagement and knowledge sharing among people and teams may represent one practical implication of the study.

Further research avenues may include the investigation of specific techniques or best practices, in the healthcare sector or different business fields, including the role of new technologies in fostering and facilitating such dynamics. 

Like any piece of research, this paper features some limitations. First, the case study investigates one specific sector. Thus, its replicability to other industries has yet to be proved. Moreover, the limited number of respondents may bias the results. We think that these limitations could be the basis for further developments of the research, enlarging the sample and applying the same methodology to other healthcare departments or different industries. Future research avenues may deepen such aspects, conducting the analysis in other sectors or locations.

## Figures and Tables

**Table 1:** Dynamic and Static Knowledge.

Dynamic Knowledge	Static Knowledge
Opinions, behaviours, ideas, and informal conversation. Workshops, communities of practice, and meetings.	Reports, memos, document procedures, databases, and other kind of organisational documentation.

Source: Sousa, MJ (2010).

**Table 2:** Findings—individual knowledge.

**Competencies**	**Description**
*Technical Competencies*	They integrate concepts about technical knowledge, including context and processes, and operational methods and means. They are the basis for the organisations’ strategic management of competencies. This kind of knowledge is easily shared because of its explicit nature.
**Application in the Case Study**	
The Healthcare Organisation does not have these competencies mapped. However, the participants in the interviews assumed the importance of developing a process of identifying the most valuable competencies for the organisation, not only technical competencies, but also organisational and social competencies, and creating some tables of competencies to identify the crucial competencies for each job position (Operation Assistants, Emergency Room Nurses, and Emergency Medical Technicians).	
Transition Process—translate into tables all the activities and tasks, and related competencies (reported in Appendix B).	
**Competencies**	**Description**
*Organisational Competencies*	They are the basis for the organization to develop beyond services and complement the technical aspects of the work. They create a sense of community, which can lead to an increase of trust and commitment by the workers that share beliefs and behavioral rules.
**Application in the Case Study**	
In the Emergency Room department, the health professionals have different visions about the healthcare organization, especially about the structure, even if they have the same perceptions about knowledge sharing. This becomes obvious when we analyse the different healthcare professionals’ opinions and thoughts. These different perspectives of the organization may be a barrier to translate individual knowledge into the organizational dimension.	
Transition Process—use of a shared language and common understandings linked to organizational culture, which is necessary to facilitate efficient communications and common understandings that focus on the essential role of trust, shared norms, and common identification.	
**Competencies**	**Description**
*Cognitive Competencies*	They integrate complex thinking skills and analytical models used in problem-solving situations, including problem identification and definition, recognition, analysis, implementation, and monitoring.
**Application in the Case Study**	
In the Emergency Room department, the healthcare professional uses processes of reflection, including individual reflection and collaborative reflection, around specific and complex problems/situations.	
Transition Process—through ongoing learning, including formal training, informal learning, observations, and discussions, as well as work experiences, the healthcare professionals develop and refine their problem-solving capabilities. They approach many problems on a daily basis, without a great deal of conscious thought about method or approach. When complex problems emerge, they recognize that they face difficulties that require collaborative problem-solving and therefore needs the team help.	
**Competencies**	**Description**
*Social Competencies*	These competencies include working habits, communication styles, leadership skills, and teamwork.
**Application in the Case Study**	
The Healthcare Organization developed teamwork competencies, communication, and informal and formal relationships by working in teams on a daily basis.	
Transition Process—the complex environment demands that problem-solving should be carried on by teams with cross-functional collaboration and interaction using social competencies to support collaborative work. Effective problem-solving includes communication and conflict resolution skills.	

## Appendix A. Interview Script

Note: This interview script is part of a larger project, so some parts do not apply to this paper:

1. Which is the main organizational knowledge that is important to frame your individual knowledge?
2. How does the organization use the worker’s individual knowledge to help solve emergency room problems?
3. Do you help to answer the everyday emergency room problems? Could you describe a situation where that has happened?
4. Which activities and related competencies are needed to perform your job?
  a) Activities
  b) Technical Competencies
  c) Organizational Competencies
  d) Cognitive Competencies
  e) Social Competencies
5. What are the procedures when a problem occurs? Could you describe a situation where there was a problem, and how you solved it?
6. Describe some situations to help promote the transition of knowledge?
7. Which types of knowledge are in transition?

## Appendix B. Tables of Competencies

**Table A1:** Emergency Room Nurses.

**Activities**
1) Direct Care
2) Indirect Patient-Centred Care
3) Personal Development
4) Writing Services
5) Non-Nursing Duties
6) Patient Assessment
7) Patient Education
**Technical Competencies**
Human development stages
Anatomy
Physiology
Pharmacology
**Organizational Competencies**
Informed consent
Handling of evidence
Mandatory reporting of child and elder abuse
Rules, norms, and internal regulations
Organisational structure
**Cognitive competencies**
Problem-solving
Critical Thinking
Emotional intelligence
**Social Competencies**
Working habits
Communication
Leadership skills
Teamwork
Empathy

**Table A2:** Emergency Medical Technicians.

**Activities**
Cardiac First Responder
Occupational First Aider
Emergency First Responder
Intermediate Life Support
**Technical Competencies**
Administration of medicines
Bleeding control
Management of burns
Splinting of suspected fractures and spinal injuries
Childbirth
Cardiopulmonary resuscitation
Semi-automatic defibrillation
Oral suctioning
Insertion of oropharyngeal and nasopharyngeal airways
Pulse oximetry
Blood glucose monitoring
Auscultation of lung sounds
Administration of medications
**Organizational Competencies**
Handling of evidence
Mandatory reporting of child and elder abuse
Rules, norms, and internal regulations
Organisational structure
**Cognitive competencies**
Problem-Solving
Critical Thinking
Emotional intelligence
Conflict resolution
**Social Competencies**
Working habits
Communication
Leadership skills
Teamwork
Empathy

**Table A3:** Operation Assistants.

**Activities**
Setting up specialised hospital equipment
Assisting physicians with the application of casts
Transporting patients
Providing routine personal care to patients
**Technical Competencies**
Anatomy
Physiology
Cognitive impairments
Nutrition
Mental health issues
Infection control
Personal care skills
Record-keeping skills
**Organizational Competencies**
Handling of evidence
Mandatory reporting of conflict situations
Rules, norms, and internal regulations
Organisational structure
**Cognitive competencies**
Problem-Solving
Critical Thinking
Emotional intelligence
Conflict resolution
**Social Competencies**
Working habits
Communication
Teamwork
Empathy
